# Targeted DNA excision in Arabidopsis by a re-engineered homing endonuclease

**DOI:** 10.1186/1472-6750-12-86

**Published:** 2012-11-13

**Authors:** Mauricio S Antunes, J Jeff Smith, Derek Jantz, June I Medford

**Affiliations:** 1 Department of Biology, Colorado State University, Fort Collins, Colorado 80523, USA; 2 Precision BioSciences, 302 East Pettigrew Street, Dibrell Building, Suite A-100, Durham, North Carolina 27701, USA

**Keywords:** Homing endonuclease, I-CreI, Targeted marker excision

## Abstract

**Background:**

A systematic method for plant genome manipulation is a major aim of plant biotechnology. One approach to achieving this involves producing a double-strand DNA break at a genomic target site followed by the introduction or removal of DNA sequences by cellular DNA repair. Hence, a site-specific endonuclease capable of targeting double-strand breaks to unique locations in the plant genome is needed.

**Results:**

We engineered and tested a synthetic homing endonuclease, PB1, derived from the I-CreI endonuclease of *Chlamydomonas reinhardtii*, which was re-designed to recognize and cleave a newly specified DNA sequence. We demonstrate that an activity-optimized version of the PB1 endonuclease, under the control of a heat-inducible promoter, is capable of targeting DNA breaks to an introduced PB1 recognition site in the genome of *Arabidopsis thaliana.* We further demonstrate that this engineered endonuclease can very efficiently excise unwanted transgenic DNA, such as an herbicide resistance marker, from the genome when the marker gene is flanked by PB1 recognition sites. Interestingly, under certain conditions the repair of the DNA junctions resulted in a conservative pairing of recognition half sites to remove the intervening DNA and reconstitute a single functional recognition site.

**Conclusion:**

These results establish parameters needed to use engineered homing endonucleases for the modification of endogenous loci in plant genomes.

## Background

The ability to genetically engineer plants has matured over the past 25 years, producing agronomic products with superior traits, and also, controversy. One source of significant objection to genetically engineered plants is the presence of antibiotic or herbicide resistance genes, frequently called ‘selectable markers’, in crops and foods [1]. The recent approval, by the Chinese Ministry of Agriculture, of field tests for transgenic rice and maize expressing the *Bacillus thuringiensis* toxin and development of herbicide resistance traits in crops has heightened concerns (*Nature Biotechnology*, **28**, 390-391, May 2010). Many of these apprehensions could be alleviated if genetically engineered plants could be produced without selectable markers. Methods to do so are largely impractical because the frequency of stably introducing genes in plants is low. An alternative approach is to use a selectable marker for the transformation process, followed by the removal of the marker gene after the transgenic plant is obtained. Previous studies have shown this to be possible [2-7].

Because the ability to target genetic modifications to specific sites in a plant genome would facilitate both plant research and the ability to better modify commercially important crop plants, many approaches have been previously tried. One approach, based on homologous recombination (HR), typically used in yeast and mammalian cells, is largely ineffective in plants. This inefficiency is widely thought to be a result of a low rate of somatic recombination in plants and the preferential repair of DNA breaks by non-homologous end-joining (NHEJ). Consequently, successful un-stimulated homologous gene integration in plants requires large-scale screening procedures and strong positive/negative selection to identify a small number of events [8,9]. Another strategy is to improve homologous gene integration in plants by over-expressing genes involved in homologous recombination. For example, Arabidopsis plants expressing the yeast *RAD54* gene, encoding a chromatin remodeling protein, increased the homologous recombination frequency one to two orders of magnitude [10]. However, the frequency of targeted transgene integration to an endogenous site is approximately 0.01% to 0.1% in plants [11].

An alternative, and more widely investigated, strategy for the targeted modification of plant genomes is the production of a DNA break at a unique chromosomal location using a site-specific endonuclease that recognizes a relatively long, and therefore unique, DNA sequence. Targeted chromosomal DNA breaks can be exploited to produce a wide range of genome modifications including targeted gene insertion [12-15], gene excision [16], and gene knock-out [17]. The effectiveness of this strategy has been demonstrated in Arabidopsis, tobacco, and maize. In these experiments, a DNA break was produced in the plant genome using a rare-cutting LAGLIDADG homing endonuclease, either the I-SceI enzyme from *S. cerevisiae*, or I-CeuI from *C. eugametos*[12,13]. Because recognition sites for these enzymes do not occur naturally in the plant genome, it was necessary, in each case, to insert an endonuclease recognition site into the genome prior to targeting it with the corresponding endonuclease. This need to “pre-engineer” plants to incorporate an endonuclease site limits the utility of natural (unmodified) homing endonucleases as genome engineering tools.

A promising alternative to natural rare-cutting endonucleases is the production of engineered DNA-cleaving enzymes that can be directed to existing, user-specified locations in a plant genome. One such approach that has garnered attention is utilization of zinc-finger nucleases (ZFNs) [18,19]. ZFNs, chimeric fusions between a zinc-finger DNA binding domain and the FokI nuclease domain, have the ability to recognize and cut existing sites in a genome because the zinc-finger domain can be engineered to recognize a variety of different DNA sequences. The power of ZFNs as genome modification reagents is highlighted by several publications in which engineered ZFNs were used to target homologous integration at native sites in the human genome [20-24]. ZFNs have also been tested in Arabidopsis, tobacco, and maize and shown to be capable of targeting mutations to introduced sites by NHEJ and HR with frequencies as high as 16% and 2%, respectively [25,26]. However, two significant limitations of ZFN are reported: (1) toxicity in plants and mammalian cells, presumed to be caused by “off-site” cleavage [27,28], and (2) imprecise events associated with their cleavage (e.g., deletions, small insertions) [29]. In addition, a similar approach to ZFNs has been obtained by fusing the FokI domain to transcription activator-like (TAL) effector proteins identified in plant pathogenic bacteria from the genus *Xanthomonas*. These TAL effector nucleases (TALEN) have been shown to successfully create targeted double-strand breaks in mammalian cells and plant protoplasts [30-32]. While the versatility of ZFNs and TALEN lies in their ability to be engineered to recognize widely divergent DNA sequences, recent publications show that this versatility can be introduced into other endonucleases. For example, protein engineering has also been applied to LAGLIDADG homing endonucleases [33-35]. These “custom” endonucleases derived from I-SceI and its homologs, I-MsoI and I-CreI, have also been shown to target DNA breaks in bacteria, yeast, and mammalian cell lines. More recently Fauser *et al.* (2012) reported a highly efficient gene targeting system in Arabidopsis that also uses a site-specific endonuclease. The improvement relies on the fact that the enzyme cuts both within the target and the chromosomal transgenic donor, leading to an excised targeting vector [36].

We report here that an engineered homing endonuclease can be used to target DNA breaks in a higher plant. To demonstrate the strength of using rationally designed homing endonucleases for plant genome engineering, we produced an endonuclease called “PB1”, derived from the natural I-CreI endonuclease, but which recognizes and cuts a very different DNA sequence. We show that this enzyme can efficiently cleave its intended recognition sequence present on a stably integrated transgene in the Arabidopsis genome. We report that optimal *in planta* cleavage requires the addition of an N-terminal nuclear localization signal and introduction of a point mutation to increase DNA cleavage activity. Lastly, we demonstrate that this optimized PB1 endonuclease can be used to efficiently excise an herbicide resistance marker from transgenic Arabidopsis plants when the marker is flanked by recognition sequences for the enzyme. These results show that rationally designed endonucleases derived from I-CreI may prove to be highly adaptable tools for plant genome engineering.

## Results

### Production and *in vitro* analysis of the PB1 endonucleases

**Figure 1:**
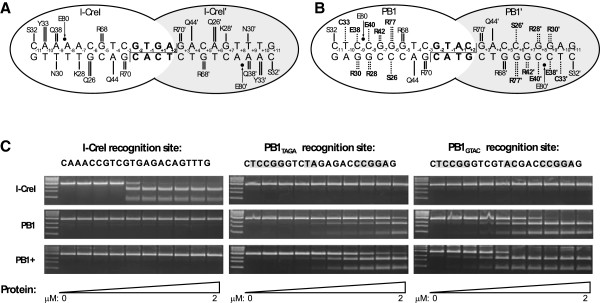
**DNA-protein interactions of endonucleases and their *in vitro *cleavage of distinct DNA substrates.** (**A**) Diagram of wild-type I-CreI homodimer in complex with its natural DNA recognition sequence. One I-CreI monomer is shown in white, the other (I-CreI’) in grey. DNA sequence is indicated, with four base-pair center sequence shown in bold. Direct hydrogen bonds between I-CreI and DNA are shown as black lines. Sites of phosphodiester bond cleavage and the resulting 4 bp 3^′^ overhangs are indicated by a line. A likely unfavorable electrostatic interaction between E80 and a backbone phosphate is indicated by a small arrow. (**B**) Predicted interactions between rationally designed PB1 endonuclease and the RS_GTAC_ DNA site. The two monomers (PB1 and PB1^′^) and DNA interactions are as indicated in (**A**), except amino acids that deviate from I-CreI and I-CreI’ hydrogen bonds (or a hydrophobic interaction, C33) and are predicted to contribute to altered DNA-cleavage specificity are indicated with dashed lines. PB1+ endonuclease contains a mutation (E80 to Q80) predicted to eliminate the unfavorable interaction mentioned in (**A**). (**C**) Cleavage of DNA by native and rationally designed endonucleases. I-CreI, PBI, and PB1+ endonucleases were incubated with three distinct linearized DNA substrates (sequence indicated above its respective set of digests). Sequence differences between I-CreI (wild-type) and the two PB1 recognition sites highlighted in grey. DNA for PB1_TAGA_ (center) and PB1_GTAC_ (right) differ from each other by the 4 bp center sequence (subscript). Digests were conducted with 0, 0.007, 0.015, 0.031, 0.062, 0.125, 0.25, 0.5, 1, 2 μM endonuclease.

The native enzyme, I-CreI, is a homodimer whose natural function is recognition and cleavage of a 22 bp DNA sequence in the *Chlamydomonas reinhardtii* chloroplast genome [37]. Figure 1A diagrams how the I-CreI protein contacts the 22 bp cleavage site. Each monomer of the homodimer makes direct and water-mediated contacts with a nine base-pair “half-site”. The two half-sites, inverted with respect to one another, are separated by a four base-pair center sequence that the endonuclease does not directly contact. The enzyme cleaves the phosphodiester bonds on either side of this center sequence, leaving two stretches of unpaired four base-pair 3^′^ DNA overhangs. Structural analyses of I-CreI in complex with a variety of DNA sites reveal that the enzyme has a relatively simple DNA recognition mechanism by which individual bases in the cleavage sequence are specified through direct contacts with a single amino acid side chain [38-40]. This mechanism lends itself to the production of engineered endonucleases with altered cleavage site preferences because, first, individual base preferences can be changed by mutating a small number (1-3) of amino acids in the enzyme, and second, the mutations that affect individual base preferences are largely independent of one another, allowing “mixing and matching” to produce endonucleases with comprehensively redesigned DNA recognition properties [34,41].

To determine whether an engineered endonuclease can specifically direct DNA cleavage to an introduced site in a plant genome, a structure-based design strategy was employed. The PB1 endonucleases were designed to recognize a nine base-pair half-site 5^′^-CTCCGGGTC-3^′^ that differs at five out of nine bases from the half-site recognized by the native I-CreI enzyme, 5^′^-CAAA(A/C)(C/T)GTC-3^′^ (bases where the two differ are underlined). Because the enzyme is a homodimer, we predict that the re-designed PB1 should recognize and cleave the 22 base-pair recognition sequence 5^′^-CTCCGGGTC-NNNN-GACCCGGAG-3, where NNNN is a highly variable four base-pair center sequence. We introduced eight amino acid changes into the endonuclease monomer in order to alter the sequence recognition of the resulting PB1 endonuclease (Figure 1B). In addition, because we previously observed that alteration of the glutamic acid residue at position 80 to glutamine (E80Q) increases the overall activity of the endonuclease without affecting its cleavage site preference, we also incorporated this change in PB1 to produce a higher activity endonuclease, referred to later in the text as PB1+.

The PB1 endonuclease variants, as well as wild-type I-CreI, were expressed in *E. coli*, purified, and evaluated *in vitro* for the ability to cleave DNA substrates containing the intended target recognition sites (RS). Figure 1C shows that the PB1 and PB1+ endonucleases efficiently cleave their intended recognition site but do not cleave the wild-type recognition site. As predicted, the PB1+ endonuclease (bottom row) cleaves its intended site more efficiently than PB1 (center row), which lacks the E80Q mutation. The crystal structure of I-CreI in complex with its preferred DNA site suggests that the center sequence does not play a major role in I-CreI recognition [38], however, some cleavage studies have indicated that certain central four base pair sequences are cut more efficiently. To test the impact of the central four base pair sequence, we compared cleavage of DNA substrates that differ only at these center four base pairs. Figure 1C shows a higher PB1 cleavage efficiency using a DNA substrate with the I-CreI consensus center sequence (5^′^-GTAC-3^′^, denoted RS_GTAC_) compared to a DNA substrate with a differing center sequence (5^′^-TAGA-3^′^, denoted RS_TAGA_).

### PB1 can cleave an introduced recognition site *in planta*

**Figure 2:**
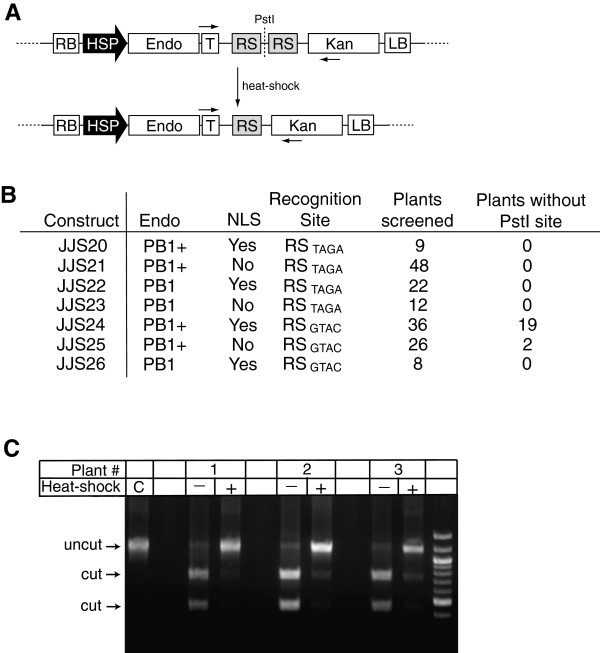
***In planta *cleavage of PB1 recognition sites by engineered endonucleases.** (**A**) T-DNA structure before and after induction of the endonuclease. Endonuclease cleavage excises the central fragment 5^′^-TTCTGCAG-3^′^, eliminating the indicated *Pst*I site. *RB*, right border; *HSP*, Hsp18.2 promoter; *Endo*, PB1 or PB1+, endonuclease; *T*, *Nopaline Synthase* terminator; *RS*, endonuclease recognition site (RS_TAGA_ or RS_GTAC_); *Kan*, kanamycin resistance marker; *LB*, left border. Horizontal arrows indicate approximate locations of PCR primers used for diagnostic evidence of *in planta* endonuclease cleavage. (**B**) Table of experimental results. Seven different T-DNA constructs used in this study, with the general form diagramed in (**A**). Each T-DNA has three possible differences: presence (Yes) or absence (No) of a nuclear localization signal (NLS) on the endonuclease; the endonuclease with either the lower activity PB1 or higher activity PB1+ (containing Q80E mutation) PB1 recognition sites (RS) contain either a TAGA or GTAC central 4 bp sequence. Plants containing some constructs (JJS20, 23, and 26) had a low recovery rate after heat shock treatment, resulting in a lower number of plants screened. (**C**) Sample agarose gel data showing loss of the *Pst*I restriction site from genomic DNA following heat-shock treatment of JJS24 plants. The agarose gel shows three JJS24 samples that demonstrated loss of the *Pst*I site. Control (**C**) shows size of uncut PCR fragment. PCR fragments from samples before heat shock (–) are cut >90% into smaller bands (identified as “cut” on left). After heat shock (+), PCR fragments from the three samples are largely uncut by *Pst*I, indicating a loss of the *Pst*I site *in planta.*

To determine the requirements for engineered endonuclease function in plants, we conducted a series of experiments using the PB1 and PB1+ endonucleases and two introduced recognition sites flanking a *Pst*I site (Figure 2A). Arabidopsis plants were individually transformed with seven different T-DNA constructs encoding the PB1 (JJS22, JJS23, and JJS26) or PB1+ (JJS20, JJS21, JJS24 and JJS25) endonucleases under the control of a heat-shock inducible promoter (Figure 2B). Distinct endonuclease and RS sites allowed us to test various aspects about function of the synthetic endonucleases in plants. First, we tested whether a nuclear localization signal (NLS) is needed for endonuclease function by including the SV40 NLS in four of these constructs (JJS20, JJS22, JJS24, and JJS26). Second, we tested the ability of the PB1 endonucleases to cleave recognition sites with the I-CreI consensus center sequence, RS_GATC_ (JJS24, JJS25, and JJS26), or distinct from the consensus sequence, RS_TAGA_ (JJS20, JJS21, JJS22, and JJS23). Third, we tested *in planta* function of the E80Q mutation (PB1 and PB1+), which is thought to provide a more favorable interaction of the endonuclease and DNA backbone.

We produced at least 20 independent primary transgenic plants (T_1_) for each distinct T-DNA. To test the function of the two PB1 enzymes and RS in plants, we induced expression of the endonucleases by subjecting plants to a heat-shock treatment and harvested individual leaves for analysis. Western blot analyses confirmed that the endonuclease was not expressed at detectable levels prior to heat shock, with expression strongly induced by the two-hour heat shock (data not shown). Genomic DNA was isolated from comparable leaves before and after induction then analyzed to determine whether the PB1 endonucleases function in plants (Figure 2B, 2C, and Additional file 1). As an initial test for PB1 function in plants, we used PCR to amplify a genomic fragment that encompasses the pair of RS and tested for the presence or absence of the *Pst*I site. If both RS are cleaved by the engineered endonuclease, an intervening fragment is excised, removing the *Pst*I site. Alternatively, cleavage of one site could produce a deletion of the *Pst*I site during non-homologous end joining repair of the break. We scored our DNA as “intact”, if greater than 90% of the amplified DNA was digested with *Pst*I, or “cleaved”, if a substantial amount of the leaf DNA (represented by greater than 30% of the amplified DNA) was resistant to *Pst*I digestion, suggesting loss of the internal fragment. We only counted samples as “cleaved” if the unheated control sample showed significant *Pst*I digestion or, in a few cases, if the unheated sample did not PCR amplify, then a sample was counted as “cleaved” only if greater than 80% were not digested by *Pst*I. In a few cases, both the heat-shocked and non-heat-shocked samples were similarly resistant to *Pst*I digestion. These samples may have integrated the endonuclease gene next to an endogenous promoter or enhancer such that the endonuclease was expressed in the absence of induction. These samples were not counted as positive results.

Genomic DNA samples isolated from all transgenic plants before PB1 induction contain the intact *Pst*I site (Figure 2B), indicating that the recognition sites are intact prior to endonuclease expression. Similarly, plant lines (JJS20, JJS21, JJS22, JJS23) containing the four base-pair center sequence (RS_TAGA_) which differs from that found in the I-CreI crystal structure, also had intact *Pst*I sites even after induction of the PB1 or PB1+ endonucleases. These results indicate that a differing four base-pair center sequence, which decreased the efficiency of the *in vitro* cleavage reaction, also hinders endonuclease function *in planta*.

We then examined whether the designed PB1 endonuclease cleaves plant DNA containing the four base-pair center sequence (RS_GTAC_) found in the crystal structure described above. Three different lines (JJS24, JJS25 and JJS26) were generated with this RS flanking the *Pst*I site. Plants were treated as described above and genomic DNA analyzed before and after induction of the endonucleases. Plant lines containing JJS26 express the PB1 endonuclease with the naturally occurring E80 residue, and upon induction of the PB1 endonuclease, the *Pst*I site is intact. In contrast, plant lines (JJS24 and JJS25) containing the PB1+ endonuclease with the Q80 mutation, lose the internal *Pst*I site after endonuclease induction (Figure 2B, 2C, and Additional file 1). These results suggest an *in planta* requirement for the favorable protein-DNA contact of Glutamine (Q) at position 80, which improves the cleavage activity of PB1+. Similarly, a need for an NLS on the engineered PB1 endonucleases is also demonstrated, whereby nineteen out of thirty-six independent transgenic plants with the NLS (JJS24) had PB1+ cleavage, compared to two out of twenty-six independent transgenic plants without the NLS (JJS25) (Figure 2B, 2C, and Additional file 1).

Genomic DNA from the PCR-amplified region both before and after induction of the endonuclease was cloned and the DNA sequence determined. All cloned fragments from non-heat-shocked plants have genomic DNA sequences that are indistinguishable from the originally introduced T-DNA (data not shown). In contrast, genomic DNA clones from the heat-shocked plants have the *Pst*I site deleted with frequencies ranging from 46% to 63% in the case of JJS24, or 49% in the case of JJS25. Unexpectedly, 100% (23 out of 23, representing eight independent transgenic plants) of the clones that lacked the *Pst*I site had a very precise deletion of the DNA sequence intervening the two RS_GTAC_ cut sites with reconstitution of a new RS_GTAC_ cut site (as drawn in Figure 2A), suggesting repair by simple re-ligation of the two cut ends. From these data, we conclude that an engineered PB1 homing endonuclease is capable of cleaving an integrated recognition site *in planta*. However, only the activity-optimized PB1+ enzyme yielded detectable cleavage of the genomic DNA site, suggesting a higher activity requirement in plants as opposed to *in vitro* assays.

### Engineered endonuclease excises a selectable marker in transgenic plants

**Figure 3:**
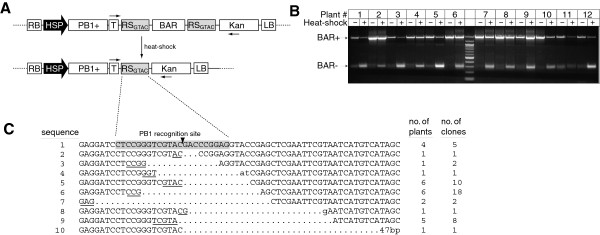
**Induction of PB1+ endonuclease removes BAR gene from Arabidopsis plants.** (**A**) Schematic of the JJS30 T-DNA before and after induction of the PB1+ endonuclease. Two RS_GTAC_ sites flank the BAR gene, so that induction of the endonuclease excises the herbicide resistance gene from the genome. The heat-inducible promoter Hsp18.2 controls expression of PB1+. Arrows indicate location of PCR primers used to assay for BAR excision. (**B**) PCR analysis of JJS30 primary transformants before and after heat-shock, using primers shown in (**A**). Unmodified JJS30 T-DNA yields a PCR product approximately 1200 bp in length (BAR+), whereas JJS30 lacking the BAR gene is approximately 300 bp (BAR–). (**C**) DNA sequence of repair junctions from BAR– clones. The approximately 300 bp PCR products from (**B**) were cloned and sequenced to evaluate the DNA repair junctions. Forty-six clones were evaluated that represented ten plants yielding a significant amount of BAR minus (-) PCR product (excluding plants 2 and 11). Ten unique sequences were obtained and these are aligned with the “perfect re-ligation” product (sequence 1), in which the reconstituted PB1 recognition site is shaded and the location of phosphodiester bond cleavage/re-ligation is indicated by the arrowhead. Total number of independent clones that yielded each sequence is indicated, as well as the number of individual transformed plants that yielded those clones. Bases that are conserved between the two halves of the repair junction (microhomology) are underlined. Single and double base insertions at the repair junction are shown in lower case (sequence 8 and 4, respectively).

To determine if the length of DNA separating a pair of PB1 recognition sequences affects the ability of the PB1 endonuclease to cleave both sites and remove the intervening sequence, we modified the JJS24 T-DNA so that the phosphinothricin acetyltransferase (BAR) gene, encoding resistance to the Basta® herbicide (under control of the Nopaline Synthase promoter), is inserted into the *Pst*I restriction site, producing JJS30 (Figure 3A). This modified T-DNA was introduced into Arabidopsis, and transgenic plants were selected for resistance to kanamycin and Basta®. We analyzed twenty-two independent T_1_ (primary transformant) plants for the presence and absence of the BAR gene before and after induction of the PB1+ endonuclease with heat shock (as described above). Figure 3B shows that genomic DNA isolated prior to heat-shock primarily yields a PCR product approximately 1200 bp in length, consistent with the original introduced T-DNA containing the BAR marker. A second prominent genomic PCR product was found in 16 of the 22 plants (first 12 shown in Figure 3B; Additional file 2) after induction of PB1+ by heat-shock. These PCR products are approximately 300 bp in length, suggesting excision of the BAR marker in the plants. For plants one, three, five and twelve, excision of the BAR gene appears to be more efficient than for the others (Figure 3B). Plants nineteen and twenty-one produced a 300 bp band in the absence of the heat shock. This uninduced BAR removal may have resulted from elevated “leaky” expression of the PB1+ endonuclease due to integration of the endonuclease gene next to a strong promoter or enhancer in the genome. Although the 300 bp band intensity appears to increase after heat shock, these samples were not counted as positive results and were not further analyzed.

To determine if the smaller PCR fragment truly represents excision of the BAR gene, we cloned this product from ten heat-shocked independent T_1_ plants and determined their DNA sequence. DNA from these ten independent T_1_ plants, representing a total of 49 sequenced clones from individual bacterial colonies, confirmed removal of the BAR gene, from between the two RS_GTAC_ sites (Figure 3C). Four independent T_1_ plants (five PCR clones) that had excised the BAR gene did so in a manner that precisely reconstituted the RS_GTAC_ site, again consistent with cleavage of the T-DNA followed by simple re-ligation (Figure 3C, and Additional file 3). The remaining plants and clones had small deletions 3-47 base pairs in length. It is also possible that there are other deletions that our cloning methodology may miss, for example, larger deletions that extend beyond the priming sites used for our PCR based analyses, or DNA breaks at non-intended sites, as was recently observed in human cells that had undergone gene therapy with engineered ZFNs [42].

Three T_1_ plants from the BAR removal experiment that showed clean excision by our PCR assay were allowed to self-fertilize, and progeny that contained the T-DNA was selected by germinating seed on medium with kanamycin. To determine if excision of the BAR gene is a genomic change that is inherited in the T_2_ progeny we “painted” leaves from each plant with the Basta® herbicide. Nineteen of these T_2_ plants, representing all three T_1_ plants, were identified as kanamycin resistant, Basta® sensitive. We excised one leaf from each plant and used PCR to confirm that they contain the JJS30 T-DNA but lack the BAR gene. Three of the nineteen plants completely lacked a BAR gene (Additional file 4). The remaining sixteen plants contained some portion of cells with an intact BAR gene that was either silenced or incorrectly identified as Basta® sensitive. These chimeric plants were not analyzed further. The PCR products obtained from the three T_2_ plants lacking the BAR gene were cloned, and eight clones resulting from each PCR product were sequenced. In clones obtained from one of these three plants, the DNA sequence is consistent with another T-DNA integration or a rearrangement during integration that mutated the BAR gene. This plant was likewise not analyzed further. In DNA from two of the three T_2_ plants, all eight clones from the same plant contained the same DNA sequence lacking the BAR gene, distinctive from the mixed sequences in leaves of induced primary transformants (T_1_ plants). However, further attempts to find T_3_ plants containing only the BAR-lacking T-DNA were unsuccessful (data not shown), indicating that excision of the BAR gene does not occur in stem cells or is an extremely rare occurrence. Also of note is that one of the two observed T_2_ plants contained a reconstituted RS_GTAC_ site.

## Discussion

Re-design of endonucleases is a powerful approach towards precise modification of plant and mammalian genomes. Seligman *et al.*[41] previously changed the I-CreI endonuclease at position C33 producing altered DNA recognition. We engineered seven changes in I-CreI to produce the PB1+ endonuclease and show that this engineered homing endonuclease is capable of targeting an introduced site within the plant genome. We report that the *in planta* cleavage of a pair of juxtaposed PB1 endonuclease recognition sites, as in the JJS24 and JJS25 constructs, results in the precise excision of the intervening DNA sequence with the reconstitution of a functional recognition site. These results are somewhat contrary to the widely-held notion that NHEJ, the dominant form of DNA repair in plants, is generally mutagenic [43]. This type of “perfect re-ligation” is not entirely without precedent, however. For example, Siebert and Puchta observed analogous excision and re-ligation using a pair of I-SceI endonuclease sites in transgenic tobacco [16]. The frequency of perfect re-ligation in these experiments was low, however, relative to the frequency of mutagenic repair [15]. Because DSB repair in plants is thought to occur primarily through a single-strand annealing (SSA) mechanism that requires short regions of homology between DNA ends at the repair junction, one possibility is that the observed perfect re-ligation was due to cleavage of one of the two recognition sites with subsequent repair by SSA (or an SSA-like mechanism) at the second site. Another possible repair mechanism may have involved cleavage at both recognition sites and subsequent re-ligation of the two “sticky” ends after loss of the intervening DNA. Our current results cannot distinguish between these two possible repair mechanisms or eliminate the possibility that some *Pst*I minus samples were produced without a need for the PB1 endonuclease. By comparing heat-shocked and non-heat-shocked samples, the data clearly demonstrate that the PB1+ endonuclease stimulates the loss of the *Pst*I site. Obtaining a single repair junction from multiple independent plants is noteworthy, especially considering that due to the experimental setup each plant cell within the leaf constitutes an independent cleavage event that could have resulted in a different repair junction outcome.

Our results with the removal of the BAR gene (Figure 3) are more consistent with current models of DNA repair in plants (reviewed in [43]; [44]). In this case, positioning the two PB1 recognition sites approximately 1 kb apart resulted in a much lower frequency of perfect re-ligation. Ninety percent of the clones sequenced from ten independent JJS30 plants exhibited additional DNA deletion from the region flanking the PB1 recognition sites and the observed deletions are decidedly non-random. Only nine unique deletions were detected in 48 sequenced clones (Figure 3C). In particular, sequences 5, 6, 7, and 9 were obtained multiple times from multiple independent plants (Additional file 3). Because the endonuclease was activated in mature plants each cell constitutes an independent cleavage and repair event. As expected, the BAR removal results were chimeric but, similarly to the *Pst*I removal results, it is interesting that the same repair junctions were found repeatedly. In each case there is a 3-5 bp “microhomology” at the junction, suggesting a SSA-like mechanism of repair (microhomologies are underlined in Figure 3C). The existence of short patches of homology at DNA repair junctions is a characteristic feature of DNA repair by SSA in plants [17,45,46] and other eukaryotes [47,48]. The number of possible repair junctions may be limited by the preference for these microhomologies.

Another significant finding is the comparison between endonuclease activity determined *in vitro* and the activity observed *in planta.* For example, we observed significant *in vitro* DNA cleavage activity by the PB1 endonuclease (Figure 1B), yet, only the more active PB1+ endonuclease had detectable function in plants. Likewise, although the RS_TAGA_ sequence could be cleaved *in vitro*, only the preferred RS_GTAC_ sequence appears to be a suitable cleavage substrate *in planta.* One possibility is that there is an “activity threshold” that an endonuclease must achieve before it is able to function *in vivo* and that this threshold is higher than what is required for *in vitro* cleavage of plasmid DNA. Interestingly, a single amino acid substitution accounts for the difference between PB1 lying below the threshold and PB1+ lying above, indicating that very minor changes can determine success or failure *in vivo*. When this threshold of activity is achieved, however, as is the case for the PB1+ endonuclease paired with the RS_GTAC_ recognition sequence, *in planta* cleavage of the recognition sequence is remarkably efficient. This “all or nothing” feature of our *in planta* cleavage results suggests that the observed differences in cleavage efficiency are not merely due to reduced endonuclease expression levels in plants. Rather, there appear to be intrinsic differences between *in vitro* and *in planta* endonuclease function that could be due to differences in environment (*e.g.,* pH or solute concentrations) or, more likely, due to differences between plasmid and genomic DNA as a cleavage substrate. The chromatin structure of plant genomic DNA is a likely factor restricting accessibility of the endonuclease to DNA, thereby reducing its efficiency *in vivo*. Several studies suggest that altering chromatin *in planta* aids HR and gene targeting [10,49,50]. In our work, the heat-shock treatment used to induce the PB1+ endonuclease is also known to alter chromatin, and may make the recognition site more accessible to the endonuclease. It is also possible that this “activity threshold” is not unique to the PB1 endonucleases and is a more general characteristic of I-CreI and engineered homing endonucleases derived from it.

Though we have undertaken great effort to replicate the *in planta* experiments reported here using wild-type I-CreI, we have been unable to obtain Arabidopsis transformants with the wild-type endonuclease gene, perhaps due to leaky expression of the endonuclease resulting in toxicity. Wild-type I-CreI is known to be highly promiscuous in its cleavage site selection and toxic to a wide range of cell types [41,51-53], and the toxicity mechanism of wild-type I-CreI may parallel the toxicity mechanism of engineered ZFNs [54]. In contrast to the wild-type I-CreI, we observed no evidence of toxicity due to expression of the PB1 or PB1+ endonucleases. All plants are phenotypically normal and healthy third-generation plants containing the endonuclease-modified JJS24 and JJS30 transgenes have been produced. Recently, we demonstrated that another engineered endonuclease successfully targets an endogenous locus in maize, generating heritable deletions at the endogenous target site [34]. However, in the present work we were unable to find T_3_ or T_4_ generation Arabidopsis plants where all the cells only contained the BAR– T-DNA (data not shown), suggesting that meganuclease activity or activity of the heat inducible promoter controlling the meganuclease in stem cells is either absent or extremely rare. T_3_ and T_4_ generation plants are chimeric for the deletions, possibly as a result of spurious activation of the heat-shock inducible promoter by some factor, such as stress, during plant growth and development. Basal levels of transcription from the heat-shock inducible promoter used in the present work (*HSP18.2*) have been reported in the literature [55], and may explain the chimeric plants obtained.

While the modification of endogenous genomic loci is one application for which this technology is being developed, the PB1+ endonuclease is a valuable tool for plant biotechnology. Excising a selectable marker, such as the herbicide gene demonstrated here, can provide advanced crops and plant systems without objectionable DNA. The significance of our achievement is demonstrated in the numerous previous efforts towards this end. For example, previous reports have described the development of site-specific recombinases for marker-gene excision (for review, see [56-59]). Zinc finger nucleases have also recently been shown to remove an intervening transgene by flanking the transgene with recognition sites [7]. It is difficult to make any comparisons with this work however, because multiple tandem recognition sites were used on both sides of the transgene. In addition, pioneering work by Puchta and coworkers has demonstrated that the I-SceI homing endonuclease can be used to excise a selectable marker gene integrated between a pair of I-SceI recognition sites in transgenic tobacco at frequencies ranging from 19 to 75% [16]. By flanking the recognition sites with a short stretch of duplicated DNA sequence, it was possible for these authors to obtain plants in which the I-SceI-induced DNA breaks were repaired through recombination between the repeated sequences. The outcome of these events was the removal of both the selectable marker and the I-SceI recognition sites from the genome. Marker gene excision using a recombinase, in contrast, necessarily leaves the recognition site(s) behind in the genome. We demonstrated that the PB1+ endonuclease is capable of catalyzing the efficient removal of a selectable marker from Arabidopsis plants in a manner analogous to I-SceI. Because it is possible to engineer a large number of I-CreI variants that recognize widely divergent DNA sequences, it should be possible to independently manipulate multiple T-DNAs and transgenes in the same plant by flanking the T-DNAs with different endonuclease recognition sites. In this study, the recognition sites for the endonuclease were introduced in order to simplify the experiments, by producing a pair of identical recognition sites flanking an easily monitored marker (*Pst*I site or BAR gene). Using the criteria learned from these experiments however, it may also be possible to modify already integrated or endogenous sequences by custom engineering an endonuclease to recognize target sites within these sequences. For example, a custom meganuclease was engineered to target an endogenous sequence in maize [34]. The design process for a custom homing endonuclease is still more complex than designing a TAL or zinc finger nuclease, but numerous groups are working to routinely generate custom meganucleases as a viable third option for genome engineering. Our system provides a clear alternative to TAL and zinc finger nucleases. Yet, given the effectiveness and ease of use of the TAL system, re-engineered homing endonucleases may have niche specific applications.

## Conclusions

The results reported here constitute a significant step toward the use of engineered homing endonucleases for the modification of endogenous loci in plant genomes. Such alterations, removing selectable markers, targeted integration of transgenes, and modification of endogenous genes may go far to reduce public objections to genetically modified plants, enhancing biotechnology’s ability to provide sustainable food and fuel resources.

## Methods

### Plant material, transgenic plant production and growth conditions

*Arabidopsis thaliana* (ecotype Col-0) was used for transformation. Plasmids were assembled as described below and transferred into *Agrobacterium tumefaciens* strain GV3101 by electroporation. Arabidopsis plants were transformed by floral dip method [60]. Primary transgenic T_1_ plants were selected on culture medium containing full-strength MS media [61], 0.8% agar, pH 5.7. Kanamycin (50 mg/L) (Sigma-Aldrich, St. Louis, MO), and/or glufosinate (5 mg/L) (Basta®; Crescent Chemical, Islandia, NY) were added to the medium as needed for the selection required for the transgenic plants. T_1_ lines were selected and allowed to self-pollinate. Single T-DNA insertion lines were identified by segregation of the Kanamycin resistance gene in the T_2_ generation. Transgenic seeds were sterilized and cold treated to synchronize germination for 1-3 days at 4°C, and were grown at 23-25°C under 16 hours light (70-100 μE.m^-2^.s^-1^ fluorescent light)/8 hours dark cycle, in either a Percival AR75L growth chamber or light shelf.

### Synthesis of the PB1 and PB1+ vectors

The PB1 endonuclease was produced using the oligonucleotide overlap extension method [62] of PCR to introduce mutations into a codon-optimized version of the I-CreI monomer. To produce PB1, we introduced eight amino acid changes: Q26S, K28R, N30R, Y33C, Q38E, S40E, T42R, and I77R. PB1+ was produced by introducing the additional mutation E80Q to PB1. As detailed in the table of Figure 2, some plant T-DNA constructs included an SV40 nuclear localization signal (sequence MAPKKKRKVI) at the N-terminus of the endonuclease. Plant T-DNA constructs were assembled in pCAMBIA2300 vector. An enhanced *CaMV35S* promoter with omega enhancer [63] and a *Nos* terminator were PCR amplified and subsequently fused to the endonuclease gene by overlapping oligonucleotide extension PCR. The full expression cassette was inserted between the *Hind*III and *Bam*HI sites of pCAMBIA2300. The pair of recognition sites with the intervening *Pst*I site was synthesized as oligonucleotides, phosphorylated with T4 polynucleotide kinase, annealed, and ligated between the *Bam*HI and *Kpn*I sites of pCAMBIA2300. The BAR expression cassette was PCR amplified from pCB302-3 [64] and inserted into the *Pst*I site of the JJS24 construct.

### Protein purification and in vitro endonuclease assay

The coding sequences for PB1, PB1+, and wild-type I-CreI were subcloned into a bacterial expression vector (pET-21a, Novagen). Both genes carried a C-terminal six-histidine tag to facilitate purification. The histidine tag was omitted from constructs expressed in plants. BL21 (DE3) cells were transformed with each plasmid and cultured on standard 2x YT medium containing 200 μg/mL ampicillin.

Protein expression was induced by addition of 1 mM IPTG after reducing the growth temperature from 37 to 22°C. Three hours after induction, the cells were pelleted by centrifugation for 10 min at 6,000 x g, and the pellets were resuspended in 1 mL binding buffer (20 mM Tris/HCl, pH 8.0, 500 mM NaCl, 10 mM imidazole) by vortexing. The cells were disrupted using 12 pulses of sonication (50% power), and the cell debris was pelleted by centrifugation for 15 min at 14,000 x g. The cell supernatant was diluted in 4 mL binding buffer and loaded onto a 200 μL nickel-charged metal-chelating Sepharose column. The column was washed with 4 mL wash buffer (20 mM Tris/HCl, pH 8.0, 500 mM NaCl, 60 mM imidazole) and then 0.2 mL elution buffer (20 mM Tris/HCl, pH 8.0, 500 mM NaCl, 400 mM imidazole). The enzymes were eluted in 0.6 mL elution buffer and concentrated to 50–130 μL using Vivaspin disposable concentrators (ISC BioExpress). The enzymes were exchanged into SA buffer (25 mM Tris/HCl, pH 8.0, 100 mM NaCl, 5 mM MgCl_2_, 5 mM EDTA) for assays and storage using Zeba spin desalting columns (Thermo Scientific). The purity and molecular weight of the enzymes were then confirmed by MALDI-TOF mass spectrometry. For in vitro cleavage assays, 25 pmol of a pUC19 plasmid harboring the meganuclease recognition sequence was linearized using *Xmn*I, then incubated with the indicated concentration of purified meganuclease for 1 h at 37°C in 10 mM Tris, pH 8.0, 50 mM NaCl, 10 mM MgCl_2_. Reactions were stopped by addition of 0.5% SDS, 25 mM EDTA and 10 μL Proteinase K (New England BioLabs). After additional 1 h incubation at 37°C, plasmid digestions were separated by gel electrophoresis, and the cut and uncut DNA bands were quantified using the ImageJ software (http://rsbweb.nih.gov/ij).

### Induction of expression of PB1 and PB1+ in plants

Transgenic T_1_ plants were selected in MS media supplemented with the appropriate selection agents as described above, and expression of the PB1 and PB1+ endonucleases was induced by heat-shock when plants were three weeks old. Heat-shock treatment consisted in submerging Parafilm-sealed plates containing plants in a water bath at 40°C for two hours, according to [50]. For genomic DNA extraction and subsequent PCR analysis, one leaf was removed prior to the heat-shock treatment and quickly frozen in liquid N_2_ (– heat-shock sample), and another leaf was removed after plants were allowed to recover from the heat-shock treatment for 24 hours (+ heat-shock sample).

### PCR and Sequence analysis of recombination events

Genomic DNA was isolated from Arabidopsis leaves using the Extract-N-Amp kit (Sigma-Aldrich) according to the manufacturer’s instructions. The region of DNA encompassing the PB1 recognition sites was PCR amplified using the primers: 5^′^-GCTCTAGCCAATACGAAACC-3^′^ and 5^′^-CTCTAGAGAAATGTTCTGGCACCTG-3^′^. For the initial set of experiments screening for the loss of a *Pst*I restriction site situated between the PB1 recognition sites, the PCR amplified fragments were digested overnight at 37°C with 20 U *Pst*I (New England BioLabs) in 1x NEB3 buffer. The digested products were resolved on a 2% agarose gel and visualized with ethidium bromide on a UV light source. For the BAR expression cassette removal experiment, the same region of the T-DNA was PCR amplified but the PCR products were directly resolved on a 1.5% agarose gel. PCR fragments corresponding to the loss of the BAR expression cassette (~300 bp) were excised from the gel and purified using QIAquick gel extraction kit (Qiagen). The purified PCR fragments were blunt-end cloned into the *Sma*I site of pUC19 vector. Colonies containing inserts in the vector were identified by blue-white screening. Plasmid DNA was isolated using Qiagen DNA mini-prep kits and sequenced using the M13R primer (5^′^-CAGGAAACAGCTATGACC-3^′^).

## Competing interests

J. Jeff Smith is Chief Science Officer and Derek Jantz is Vice-President of Scientific Development at Precision BioSciences. June Medford is in the Advisory Board of Precision BioSciences.

## Authors’ contributions

JJS participated in the design of the study, carried out part of the experimental work, drafted the manuscript, and obtained funding. MSA participated in the design of the study, carried out part of the experimental work, and contributed to manuscript drafting and revising. DJ participated in the design of the study and obtained funding. JIM was overall study supervisor, participated in study design, helped write the manuscript and obtained the funding. All authors read and approved the final version.

## Supplementary Material

Additional file 1 **Figure S1.***In planta* cleavage of PB1 recognition sites by engineered endonucleases following heat-shock, resulting in loss of *Pst*I site. Agarose gel shows a *Pst*I screen of the remaining thirty two JJS24 samples before and after heat shock. PCR fragments from samples before heat shock (–) are cut > 90% into product bands (identified as “*Pst*I cut PCR” on right side of gel). After heat shock (+), the PCR fragments from the three samples are largely uncut by *Pst*I, indicating a loss of the *Pst*I site *in planta*. Plant samples that demonstrated a significant resistance to cleavage by *Pst*I after heat-shock are indicated with a “*”. Sequence analysis of these cloned PCR fragments (*) confirmed the loss of the *Pst*I site and reconstitution of a single PB1 recognition site. Click here for file

Additional file 2 **Figure S2.** Induction of the PB1+ endonuclease removes the BAR gene from Arabidopsis plants. The two gels show the PCR analysis of all twenty four JJS30 transformants. Genomic DNA samples were taken from twenty four JJS30 transformants (first twelve represented in Figure 3B) before and after heat-shock, and evaluated by PCR using the primers shown in Figure 3A. The unmodified JJS30 T-DNA is expected to yield a PCR product approximately 1200 bp in length (BAR+ arrow), whereas JJS30 lacking the BAR gene is expected to be approximately 300 bp (BAR– arrow). Click here for file

Additional file 3 **Table S1.** DNA sequences of individual clones containing PCR-amplified repair junctions from ten different plants following BAR expression cassette removal. Click here for file

Additional file 4 **Figure S3.** Analysis of BAR removal in T_2_ generation arising from heat-shocked JJS30 T_1_ Arabidopsis plants. Following heat-shock and recovery, T_1_ (primary transformants) Arabidopsis plants were allowed to self-pollinate. The resulting progeny were grown on medium with kanamycin to select for the JJS30 T-DNA and screened for Basta® resistance by painting a leaf with Basta®. Genomic DNA was extracted from plants that appeared to be Basta® sensitive and the region encompassing the BAR expression cassette was amplified by PCR. PCR fragments were resolved on a 1.5% agarose gel looking for homogeneous BAR minus T-DNA. Samples 8, 15, and 19 appear to lack a copy of the BAR cassette. Samples 6, 7, 14, and 16 appear to have an equal mixture of T-DNAs with and without the BAR cassette. These samples may contain two T-DNAs or may have resulted from BAR removal in the T_1_ generation by leaky expression of the PB1+ endonuclease. Finally, samples 1, 2, 4, 5, 9, 10, 11, 12, 13, 17, and 18 appear to only contain an intact BAR cassette. These plants may have been incorrectly identified as sensitive with our Basta® painting screen, and/or they may have silenced expression of the BAR gene. The PCR fragments from samples 8, 15, and 19 were cloned and eight individual clones for each sample were sequenced to determine if they are truly homogeneous. In each case, all eight clones had the same sequence, indicating that the plants are not chimeric, unlike their parental T_1_ plants. Sample 8 had a small insertion and deletion at the repair junction. Sample 15 had a conservative repair junction with a reconstituted recognition site. Sample 19 appears to be a recombination event with another T-DNA. (PDF 1014 kb) Click here for file
