# Supplementary figures and images for: Targeted DNA excision in Arabidopsis by a re-engineered homing endonuclease
Source: BMC Biotechnol. 2012 Nov 13;12:86. doi: 10.1186/1472-6750-12-86 (PMC3536558; doi:10.1186/1472-6750-12-86)

Supplemental Figure 1.

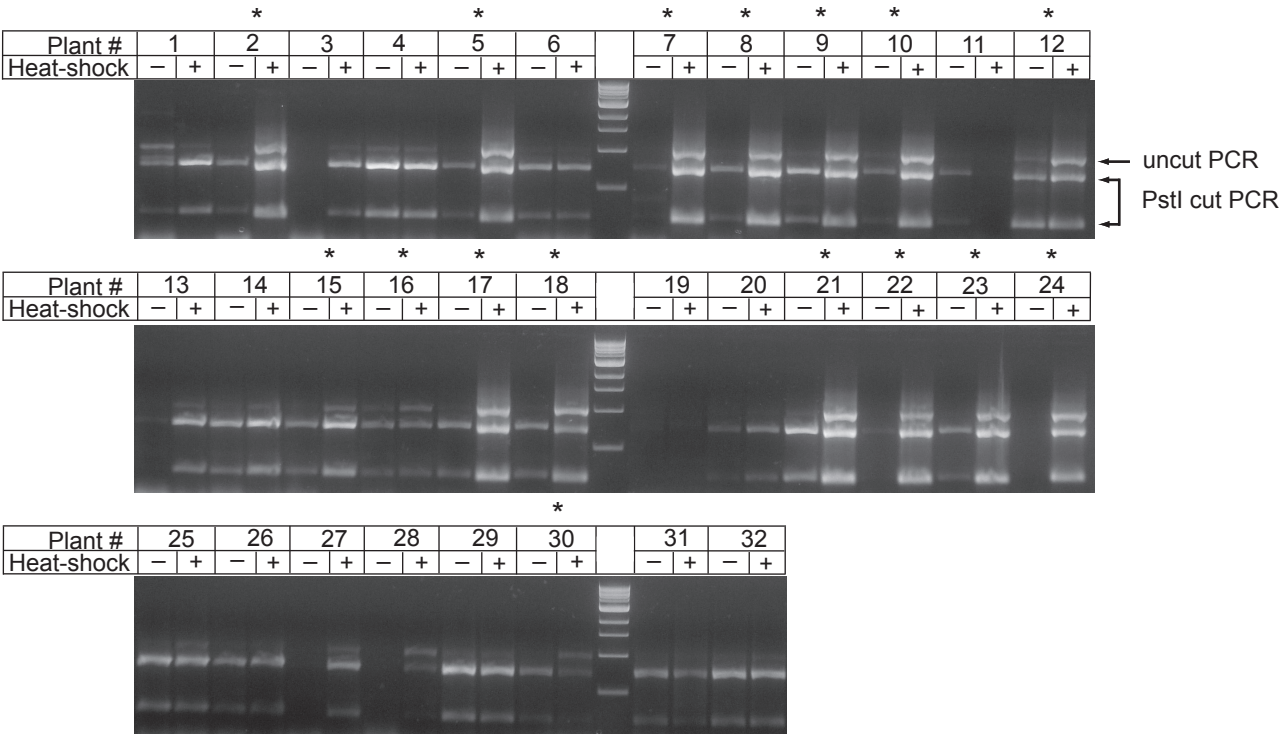

Supplement: Additional file 1 — Figure S1.In planta cleavage of PB1 recognition sites by engineered endonucleases following heat-shock, resulting in loss of PstI site. Agarose gel shows a PstI screen of the remaining thirty two JJS24 samples before and after heat shock. PCR fragments from samples before heat shock (–) are cut > 90% into product bands (identified as “PstI cut PCR” on right side of gel). After heat shock (+), the PCR fragments from the three samples are largely uncut by PstI, indicating a loss of the PstI site in planta. Plant samples that demonstrated a significant resistance to cleavage by PstI after heat-shock are indicated with a “*”. Sequence analysis of these cloned PCR fragments (*) confirmed the loss of the PstI site and reconstitution of a single PB1 recognition site. [file 1472-6750-12-86-S1.pdf]

Supplemental Figure 2.

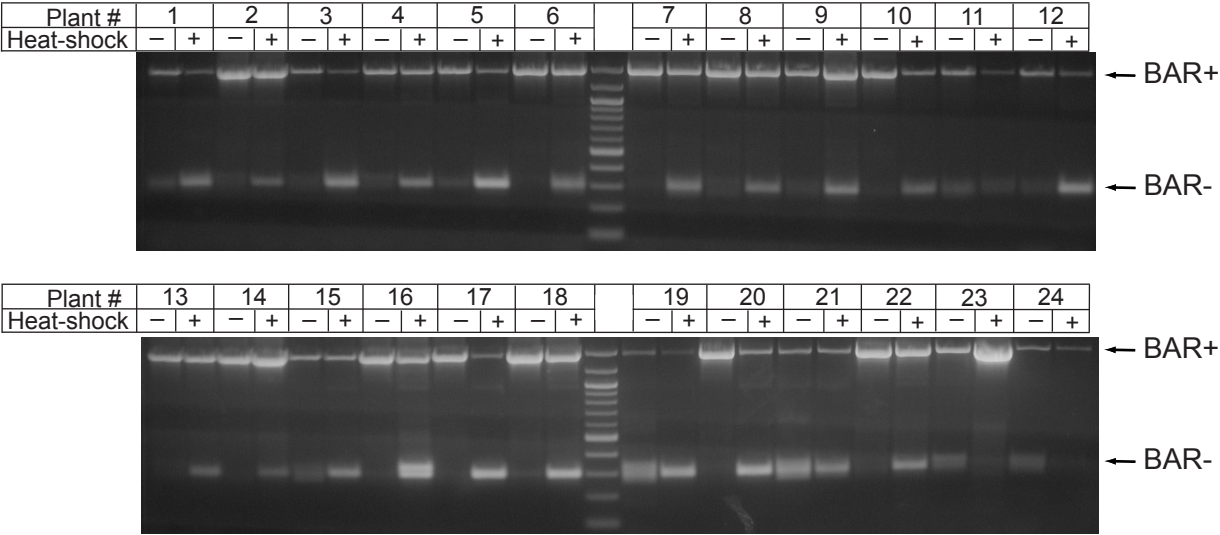

Supplement: Additional file 2 — Figure S2. Induction of the PB1+ endonuclease removes the BAR gene from Arabidopsis plants. The two gels show the PCR analysis of all twenty four JJS30 transformants. Genomic DNA samples were taken from twenty four JJS30 transformants (first twelve represented in Figure 3B) before and after heat-shock, and evaluated by PCR using the primers shown in Figure 3A. The unmodified JJS30 T-DNA is expected to yield a PCR product approximately 1200 bp in length (BAR+ arrow), whereas JJS30 lacking the BAR gene is expected to be approximately 300 bp (BAR– arrow). [file 1472-6750-12-86-S2.pdf]

Supplemental Figure 3.

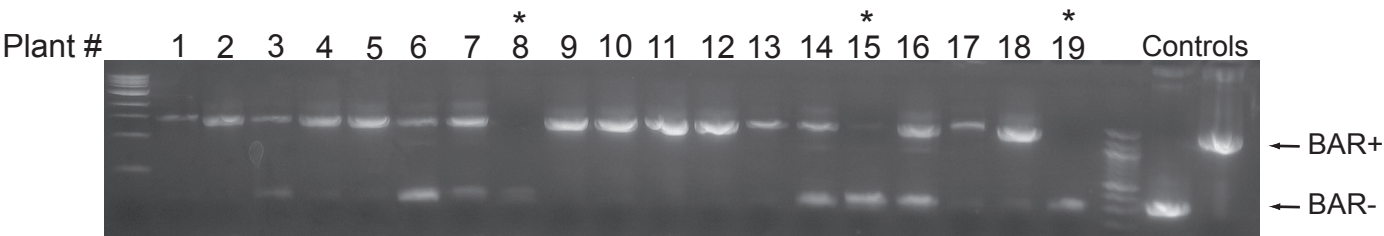

Supplement: Additional file 4 — Figure S3. Analysis of BAR removal in T2 generation arising from heat-shocked JJS30 T1 Arabidopsis plants. Following heat-shock and recovery, T1 (primary transformants) Arabidopsis plants were allowed to self-pollinate. The resulting progeny were grown on medium with kanamycin to select for the JJS30 T-DNA and screened for Basta® resistance by painting a leaf with Basta®. Genomic DNA was extracted from plants that appeared to be Basta® sensitive and the region encompassing the BAR expression cassette was amplified by PCR. PCR fragments were resolved on a 1.5% agarose gel looking for homogeneous BAR minus T-DNA. Samples 8, 15, and 19 appear to lack a copy of the BAR cassette. Samples 6, 7, 14, and 16 appear to have an equal mixture of T-DNAs with and without the BAR cassette. These samples may contain two T-DNAs or may have resulted from BAR removal in the T1 generation by leaky expression of the PB1+ endonuclease. Finally, samples 1, 2, 4, 5, 9, 10, 11, 12, 13, 17, and 18 appear to only contain an intact BAR cassette. These plants may have been incorrectly identified as sensitive with our Basta® painting screen, and/or they may have silenced expression of the BAR gene. The PCR fragments from samples 8, 15, and 19 were cloned and eight individual clones for each sample were sequenced to determine if they are truly homogeneous. In each case, all eight clones had the same sequence, indicating that the plants are not chimeric, unlike their parental T1 plants. Sample 8 had a small insertion and deletion at the repair junction. Sample 15 had a conservative repair junction with a reconstituted recognition site. Sample 19 appears to be a recombination event with another T-DNA. (PDF 1014 kb) [file 1472-6750-12-86-S4.pdf]
