# Supplementary material for: Targeted DNA excision in Arabidopsis by a re-engineered homing endonuclease
Source: BMC Biotechnol. 2012 Nov 13;12:86. doi: 10.1186/1472-6750-12-86 (PMC3536558; doi:10.1186/1472-6750-12-86)
Supplement: Additional file 3 — Table S1. DNA sequences of individual clones containing PCR-amplified repair junctions from ten different plants following BAR expression cassette removal. [file 1472-6750-12-86-S3.pdf]

Supplementary Table 1. DNA sequences of individual clones containing PCR-amplified repair junctions from ten different plants following BAR expression cassette removal.

DNA Sequence	Plant Number	Number of Clones
GAGGATCCTCCGGGTCGTACGACCCGGAGGTACCGAGCTCGAATTCGTAATCATGT	1	2
GAGGATCCTCCGGGTCG……………………………………...GAATTCGTAATCATGT	1	3
GAGGATCCTCCGGGTCGTAC………………………CGAGCTCGAATTCGTAATCATGT	1	5
GAGGATCCTCCGGGTCGTAC………………………CGAGCTCGAATTCGTAATCATGT	1	6
GAG…………………………………………………………CTCGAATTCGTAATCATGT	1	7
GAGGATCCTCCG………………………………………...AGCTCGAATTCGTAATCATGT	2	1
GAGGATCCTCCG………………………………………...AGCTCGAATTCGTAATCATGT	2	2
GAGGATCCTCCGGGTCGTA…………………………………………………..ATCATGT	2	3
GAGGATCCTCCG………………………………………...AGCTCGAATTCGTAATCATGT	2	4
GAGGATCCTCCG………………………………………...AGCTCGAATTCGTAATCATGT	2	6
GAGGATCCTCCG………………………………………...AGCTCGAATTCGTAATCATGT	2	8
GAGGATCCTCCGGGTCGTAC………………………CGAGCTCGAATTCGTAATCATGT	5	1
GAGGATCCTCCGGGTCGTA…………………………………………………..ATCATGT	5	3
GAGGATCCTCCGGGTCGTAC………………………CGAGCTCGAATTCGTAATCATGT	5	4
GAGGATCCTCCGGGTCGTA…………………………………………………..ATCATGT	5	5
GAGGATCCTCCGGGTCGTAC…………………………………………………………47bp	5	8
GAGGATCCTCCG………………………………………...AGCTCGAATTCGTAATCATGT	8	1
GAGGATCCTCCG………………………………………...AGCTCGAATTCGTAATCATGT	8	2
GAGGATCCTCCG………………………………………...AGCTCGAATTCGTAATCATGT	8	3
GAGGATCCTCCG………………………………………...AGCTCGAATTCGTAATCATGT	8	5
GAGGATCCTCCG………………………………………...AGCTCGAATTCGTAATCATGT	8	7
GAGGATCCTCCG………………………………………...AGCTCGAATTCGTAATCATGT	8	8
GAGGATCCTCCGG……………………….AGGTACCGAGCTCGAATTCGTAATCATGT	9	1
GAGGATCCTCCGGGTCGTA…………………………………………………..ATCATGT	9	3
GAGGATCCTCCGG……………………….AGGTACCGAGCTCGAATTCGTAATCATGT	9	4
GAGGATCCTCCGGGTCGTAC……CCGGAGGTACCGAGCTCGAATTCGTAATCATGT	9	5
GAGGATCCTCCGGGTCGTAC………………………CGAGCTCGAATTCGTAATCATGT	9	7

GAGGATCCTCCGGGTCGTACGACCCGGAGGTACCGAGCTCGAATTCGTAATCATGT	9	8
GAGGATCCTCCG……………………………………...AGCTCGAATTCGTAATCATGT	10	2
GAGGATCCTCCGGGTCGTACGACCCGGAGGTACCGAGCTCGAATTCGTAATCATGT	10	3
GAGGATCCTCCG……………………………………...AGCTCGAATTCGTAATCATGT	10	3*
GAGGATCCTCCGGGTCGTACGACCCGGAGGTACCGAGCTCGAATTCGTAATCATGT	10	4
GAGGATCCTCCGGGTCGTA…………………………………………………..ATCATGT	10	5
GAGGATCCTCCGGGTCGTA…………………………………………………..ATCATGT	11	4
GAGGATCCTCCGGGTCGTAC………………………CGAGCTCGAATTCGTAATCATGT	11	5
GAGGATCCTCCGGGTCGTA…………………………………………………..ATCATGT	11	6
GAGGATCCTCCGGGTCGTACGACCCGGAGGTACCGAGCTCGAATTCGTAATCATGT	11	7
GAGGATCCTCCGGGTCGTA…………………………………………………..ATCATGT	11	8
GAGGATC……………………………………...TACCGAGCTCGAATTCGTAATCATGT	15	1
GAGGATCCTCCGGGT……………………………..atCGAGCTCGAATTCGTAATCATGT	15	3
GAGGATCCTCCG……………………………………...AGCTCGAATTCGTAATCATGT	20	2
GA……………………………………………………….GCTCGAATTCGTAATCATGT	23	3
GAGGATCCTCCGGGTCGTAC………………………CGAGCTCGAATTCGTAATCATGT	23	4
GAGGATCCTCCGGGTCGTAC………………………CGAGCTCGAATTCGTAATCATGT	23	5
GAGGATCCTCCGGGTCGTAC………………………CGAGCTCGAATTCGTAATCATGT	23	6
